# Quantifier comprehension is linked to linguistic rather than to numerical skills. Evidence from children with Down syndrome and Williams syndrome

**DOI:** 10.1371/journal.pone.0199743

**Published:** 2018-06-27

**Authors:** Sarah Dolscheid, Martina Penke

**Affiliations:** Department for Special Education and Rehabilitation, University of Cologne, Cologne, Germany; Cardiff University, UNITED KINGDOM

## Abstract

Comprehending natural language quantifiers (like *many*, *all*, or *some*) involves linguistic and numerical abilities. However, the extent to which both factors play a role is controversial. In order to determine the specific contributions of linguistic and number skills in quantifier comprehension, we examined two groups of participants that differ in their language abilities while their number skills appear to be similar: Participants with Down syndrome (DS) and participants with Williams syndrome (WS). Compared to rather poor linguistic skills of individuals with DS, individuals with WS display relatively advanced language abilities. Participants with WS also outperformed participants with DS in a quantifier comprehension task while number knowledge did not differ between the two groups. When compared to typically developing (TD) children of the same mental age, participants with WS displayed similar levels regarding quantifier abilities, but participants with DS performed worse than the control group. Language abilities but not number skills also significantly predicted quantifier knowledge in a linear regression analysis, stressing the importance of linguistic abilities for quantifier comprehension. In addition to determining the skills that are relevant for comprehending quantifiers, our findings provide the first demonstration of how quantifiers are acquired by individuals with DS and WS, an issue not investigated so far.

## Introduction

*Many* a true word is spoken in jest. *Some* like it hot. And: *All* you need is love. Quantifiers like *many*, *some* or *all* are frequently used in language. However, in order to properly comprehend quantifiers, various skills are required. For instance, if a person confesses that she ate *all* of the cookies, semantic knowledge of the term *all* is needed to infer that none of the cookies will be left for you. What is more, if a person confesses that she ate *some* cookies, this entails she did not eat *all* of the cookies (otherwise the utterance would violate the pragmatic principle of informativeness, [1]). A subset of quantifiers also refers to specific quantities. If a person confesses she ate *both* cookies, you know that she ate exactly two (not three, four, or any other number). Thus, in order to successfully comprehend quantifiers, one has to know their semantic and pragmatic restrictions as well as their quantificational meanings (i.e., the specific quantity or number(-range) that is denoted by a quantifier).

Although quantifier comprehension seems to involve both linguistic and numerical processes, the extent to which both factors play a role has led to a controversy: Whereas some researchers have stressed the importance of language abilities in quantifier comprehension [2,3], others have considered numerical skills more relevant [4–8] . For instance, the same processes seem to be involved when people compare numbers or magnitudes but also when they judge the truth value of sentences containing quantifiers (such as *“Many of the circles are yellow”*) [7]. These findings suggest a close connection between number skills and quantifier comprehension. In the same vein, patients with corticobasal degeneration are not only impaired in number processing but likewise show deficits in quantifier comprehension [5]. Comprehending quantifiers also seems to recruit brain areas that subserve number comparisons (i.e., the intraparietal sulcus (IPS) [8,9]. Other studies, however, report brain activations associated with quantifier comprehension in regions for general semantic processing [10]. Inconsistent findings have also been obtained in patients with semantic dementia (i.e. the inability to understand word meanings [11]): While some patients with semantic dementia were selectively impaired in quantifier processing with no simultaneous problems in number skills [2], others displayed spared comprehension of both numbers and quantifiers [12], emphasizing links between number and quantifier knowledge.

The controversy about numerical versus linguistic contributions to quantifier comprehension also pertains to developmental studies. On the one hand, children’s quantifier skills have been found to be linked to advanced cardinal number knowledge [13] as well as to higher levels of approximate number skills [14], demonstrating close connections between quantifier and numerical abilities. On the other hand, conflicting results have been reported for children with Specific Language Impairment (SLI, i.e. selective difficulties regarding language acquisition in the absence of neural, cognitive, psycho-emotional, or sensory impairments). SLI children performed lower on a quantifier comprehension task compared to age-matched peers [3], suggesting that linguistic rather than numerical skills may be essential for the acquisition of quantifiers. However, since children with SLI do not only exhibit impaired linguistic abilities but also concomitant deficits in number skills [15,16], it remains unclear whether SLI children’s difficulties in quantifier comprehension are due to linguistic or numerical disabilities.

In order to approach the controversial issue of linguistic vs. numerical contributions to quantifier acquisition, we sought to investigate two groups of participants that differ quite substantially in their language skills while their numerical abilities appear to be comparable: individuals with Down syndrome and individuals with Williams syndrome. Down syndrome (DS) is a genetic disorder caused by a third copy of chromosome 21. Williams syndrome (WS) is a genetically based neurodevelopmental disorder caused by a microdeletion of genes from regions of chromosome 7 [17]. Despite some variation in both populations, most individuals with DS and WS display mild to moderate intellectual disabilities [18,19]. Both groups also show poor number and arithmetic skills that are not at the level of their chronological age but rather commensurate with their mental abilities [20–22]. However, whereas individuals with WS display quite advanced language abilities [23,24], linguistic skills are rather poor in individuals with DS [23,25,26]. In particular, participants with WS have been found to outperform participants with DS in a variety of language production and comprehension experiments [18], leading Bellugi and colleagues to the conclusion that “language is a major strength in WMS [Williams syndrome] and radically different from typical DNS [Down syndrome]” [18], p.11.

The difference in WS and DS participants’ cognitive profiles (i.e. similar number skills but different language abilities) makes it possible to tease apart linguistic and numerical contributions to quantifier comprehension. If number skills are indeed essential for comprehending quantifiers, quantifier comprehension should be at a comparable level for children and adolescents with DS and WS. If, on the other hand, linguistic skills are more important for quantifier comprehension, participants with WS are expected to outperform participants with DS due to their advanced language abilities. To distinguish between these possibilities, we assessed language skills, number knowledge and quantifier comprehension in children with WS and DS as well as in typically developing (TD) children. In addition to determining the abilities that are relevant for comprehending quantifiers, the current study also provides the first investigation of quantifier skills in children and adolescents with DS and WS, an issue which has not been investigated so far.

## Methods

### Participants

Fifteen monolingual German-speaking children and adolescents with DS (7 female, 8 male, mean chronological age: 10;09 years, *SD* = 3;06 years) and 10 monolingual German-speaking children and adolescents with WS (6 female, 4 male, mean chronological age:12;06 years, *SD* = 3;01 years) participated in the study. Participants were recruited via local networks as well as the German Association for Williams Syndrome. Participants were included in the study if they were monolingual speakers of German and if oral language was their primary means of communication, which was true of all of the participants examined. Individuals with a hearing loss exceeding 25 dB were not included in the study. The testing of 4 additional participants with DS could not be completed due to substantial hearing problems (*n* = 1) or the inability to comprehend the experimental tasks (*n* = 3). All participants lived with their families and either attended regular school classes or schools for children with special educational needs. Participants’ nonverbal cognitive development was assessed by the reasoning subscale of the Snijders-Oomen Nonverbal Intelligence Test (SON-R 2½-7) [27]. The mean nonverbal mental age of DS participants was 4;03 years (*SD* = 0;11 years) and the mean mental age of WS participants was 5;03 years (*SD* = 0;11 years). While the average chronological age did not differ between participants with DS and WS, *t*(23) = 1.29, *ns*, the two groups differed significantly with respect to their mental age, *t*(23) = 2.96, *p* = .007. To elucidate whether the performance of participants with DS or WS regarding number skills, verbal abilities and quantifier comprehension conformed to their nonverbal cognitive development, we compared their performance to two samples of typically developing (TD) children. TD children were part of a larger cohort (n = 41) tested independently on links between quantifier knowledge and approximate number skills (see results in [14]). We included those TD children whose mental age was on par with mental ages obtained for participants with DS or WS. In particular, a group of 15 TD children (CG_DS_) was individually matched on nonverbal mental age to the participants of the DS group (mean chronological age: 4,02 years, *SD* = 0;09 years; mean nonverbal mental age: 4;03 years, *SD* = 0;10 years). Another group of 10 TD children (CG_WS_) was individually matched on nonverbal mental age to the participants of the WS group (mean chronological age: 5;00 years, *SD* = 0;08 years; mean nonverbal mental age: 5;03 years, *SD* = 0;11 years). Three of the TD children served as controls for both the WS and DS participants, resulting in 22 TD children in total. All of the TD children were monolingual speakers of German and they were recruited from local day care centers. Children displayed no evidence of physical or cognitive impairments and none of the children had a history of hearing impairments. Vision was normal or otherwise corrected to normal. All participants were compensated for their participation by a little gift. Informed written consent was obtained from all parents or caretakers. The study was approved by Cologne University’s Medical Ethics committee and the data were collected and treated according to the ethical guidelines of the declaration of Helsinki. An overview of the participants can be found in Table 1.

**Table 1:** Overview of participants.

Group	N	sex	chronological age in years (y;mm)	nonverbal mental age in years (y;mm)^a^
DS	15	7f, 8m	*M* 10;09 *SD* 3;06 range 6;06–16;07	*M* 4;03 *SD* 0;11 range 3;04–5;08
CG_DS_	15	7f, 8m	*M* 4;02 *SD* 0;09 range 3;03–5;09	*M* 4;03 *SD* 0;10 range 3;04–5;08
comparison between groups (independent-samples t-test)			*p*< .001	*p* = .97
WS	10	6f, 4m	*M* 12;06 *SD* 3;01 range 6;06–16;08	*M* 5;03 *SD* 0;11 range 4;04–7;01
CG_WS_	10	4f, 6m	*M* 5;00 *SD* 0;08 range 4;01–6;01	*M* 5;03 *SD* 0;11 range 4;04–7;04
comparison between groups (independent-samples t-test)			*p*< .001	*p* = .96

DS = participants with Down syndrome, CG_DS_ = Control Group of TD children matched on mental age to participants with DS, WS = participants with Williams syndrome, CG_WS_ = Control Group of TD children matched on mental age to participants with WS.

^a^Participants’ nonverbal mental age was determined by the reasoning subscale of the Snijders-Oomen Nonverbal Intelligence Test (SON-R 2½-7) [27].

### Materials and procedure

Each participant was tested individually on three tasks: 1) a task assessing number knowledge (i.e. the Give-*n* task), 2) a standardized task examining language skills (*Sprachscreening fuer das Vorschulalter* (SSV), ‘language screening for preschool children’) [28], and 3) a task examining quantifier knowledge (i.e. the Give-quantifier task). The tasks were presented on three different sessions and were part of a larger testing battery examining other numerical and spatial skills in typically and atypically developing children. To avoid spill-over effects due to task similarities, the order of tasks was fixed: During the first session, the Give-quantifier task and the language screening were administered. In the second session, the Give-*n* task followed. Children’s nonverbal mental age was assessed by the SON-R 2½-7 [27] during the third session.

#### The Give-n task

To examine children’s number knowledge, the Give-*n* task was adapted from Wynn [29], also see [14]. Children were first introduced to a glove puppet called “Tillman the Dog”. Participants were then asked to ‘feed’ the dog by putting a specific number of lemons into a white plastic bowl. Stimuli consisted of eight plastic lemons. Requests were of the form: “Kannst du dem Hund *n* Zitronen geben?” “Can you give the dog *n* lemons?” A titration method was used [29]: Participants were first asked for one item and subsequently for three items. Further requests depended on children’s previous responses. In case participants responded correctly to a request for N (e.g. n = 3), they were then asked for N+1 (e.g. n = 4). However, when children responded incorrectly to a request for N, they were subsequently tested on N-1 (e.g. 2). The highest number requested was “6”. Children were classified as N-knowers (e.g., three-knowers) if they correctly gave N lemons two out of three times but failed to give the correct number two of three times for N+1. Participants who had at least twice as many successes as failures for trials of five and six were classified as cardinal principle-knowers (CP-knowers). That is, once children grasp the principle of cardinality they know that the last word in a counting sequence reveals the cardinality of the whole set [29,30].

#### Language screening (SSV)

We administered the German ‘Sprachscreening fuer das Vorschulalter’ (SSV) [28], a standardized screening instrument developed to examine 3- to 5-year-old children’s linguistic skills. Since more than half of the DS participants (as well as the corresponding control group) yielded a mental age of only three years, the SSV version for 3-year-olds was used for all participating children in order to keep results comparable. The SSV consists of two subtests assessing the phonological working memory for non-words and the application of morphological rules. These subtests have been argued to measure critical aspects of language acquisition and accordingly allow for a valid determination of a child’s level of language development [28]. In the subtest assessing phonological working memory, children were asked to immediately repeat spoken non-words (e.g. *billop* or *defsal*). This test consists of thirteen items that display an increasing complexity with regard to the number of syllables (from two to five syllable words) and with respect to syllable complexity (i.e. the number of consonants in onset and offset positions). Evaluations of children’s responses were performed according to the manual of the SSV [28]. Only the exact same forms produced by the child were evaluated as correct. Following the manual, a raw score for each participant was then computed by counting the absolute number of correctly repeated non-words. In the second subtest, children were presented with ten two-part images displaying a single object on the left (e.g. a car) and multiple exemplars of the same object on the right side (e.g. three cars). Participants were asked to produce the plural form of the objects. In case participants did not produce a plural form as a response, the answer was coded as wrong (zero points). An incorrect plural form (e.g. *Gabel*s* ‘forks’) was assigned one point and the correct plural form was counted as two points, resulting in a maximum score of twenty points for this test. Raw values of the two subtests were added for each participant, resulting in an overall performance score (maximum score to be achieved = 33).

#### The Give-quantifier task

The Give-quantifier task was an adaptation from Barner and colleagues [13] (for the same procedure also see [14]). Stimuli consisted of three sets of small plastic fruits (i.e., 8 bananas, 8 oranges, and 8 strawberries) and a white plastic bowl. Sets were presented in separate piles organized by kind. The experimenter first ensured that participants knew the various fruit types by asking questions like “Do you know what this is?” or “Can you tell me what these are?”. Once participants were able to correctly name each type of fruit, the experimenter explained the task. During each trial, the experimenter pointed to the empty bowl and asked the child to put a quantity of a particular kind of fruit into it (e.g. “Kannst du *viele* von den Orangen in die Schüssel legen?” “Can you put *many* of the oranges into the bowl?”). Quantifier comprehension was assessed for the following seven quantifiers: *alle* (all), *eine* (a), *keine* (none), *die beiden* (both), *die meisten* (most), *viele* (many), and *einige* (some). All quantifiers were used in the partitive construction (e.g. many *of the* Xs). After each trial, all fruit items were returned to their original piles. Three different orders of quantifiers were administered between participants, with pairings of quantifiers and fruit kinds quasi-randomized. Each quantifier was tested three times, resulting in 21 trials in total per participant. For each participant, an overall quantifier score ranging from 0 to 3 was calculated. The score was defined as the average number of correct responses a participant made for each quantifier over three test trials. Since there are a number of different ways how one could correctly respond to vague quantifiers like *many* or *some*, we took German adults’ response ranges as a basis to evaluate children’s responses [14]. Thus, quantifier comprehension of DS, WS, and TD children was considered ‘correct’ when the range of given fruit tokens was shared by 100% of the adult German-speaking participants. Table 2 displays the correct number of items for each quantifier on the basis of adult responses.

**Table 2:** Definitions of ‘correct’ (i.e., adult-like) responses for each quantifier (maximum number of tokens = 8).

Quantifier	Correct response
*alle* (all)	8
*keine* (none)	0
*eine* (a)	1
*Die beiden* (both)	2
*Die meisten* (most)	5–8
*viele* (many)	3–8
*einige* (some)	2–6

## Results

An overview of the descriptive results obtained in the three experimental tasks is presented in Table 3 (also see S1 File).

**Table 3:** Mean scores and standard deviations of the experimental tasks.

Participant groups				
	DS	CG_DS_	WS	CG_ws_
**Number knowledge** **(maximum score = 6)**	*Mean*: 5.0 *SD*: 1.3	*Mean*: 4.3 *SD*: 1.9	*Mean*: 5.5 *SD*: 1.1	*Mean*: 6.0 *SD*: 0.0
**Language screening** **(maximum score = 33)**	*Mean*: 18.0 *SD*: 8.9	*Mean*: 23.3 *SD*: 5.3	*Mean*: 27.7 *SD*: 4.0	*Mean*: 26.9 *SD*: 4.8
**Quantifier comprehension** **(maximum score = 3)**	*Mean*: 2.2 *SD*: 0.4	*Mean*: 2.4 *SD*: 0.3	*Mean*: 2.7 *SD*: 0.2	*Mean*: 2.6 *SD*: 0.3

### Number knowledge

#### Comparison WS-DS

Analyses of the Give-*n* task revealed that 8 of 10 children with WS (80%) and 9 of 15 children with DS (60%) attained the highest number-knower level (i.e. CP-knower). The average number-knower level was 5.0 for participants with DS and 5.5 for participants with WS. When differences in mental age were controlled (i.e. entered as a covariate), the average number-knower level did not differ statistically between the two groups, suggesting a comparable level of number knowledge, *F*(1, 22) = 0.00, *ns*, *η*_*p*_^2^ = .00.

#### Comparison DS-CG_DS_

60% of the children with DS and 53% of the TD children attained the highest number-knower level in the Give-*n* task. The average number-knower level was 5.0 for participants with DS and 4.3 for mental age-matched controls. Number knowledge did not differ statistically between DS and TD children, which can be interpreted as a comparable level of number knowledge in the two groups, *t*(25) = 1.1, *ns*, Cohen’s *d* = .42.

#### Comparison WS-CG_WS_

80% of the children with WS and all of the TD children (100%) attained the highest number-knower level, with a mean number-knower level of 5.5 for participants with WS and 6.0 for mental age-matched controls. There was no statistical difference between the average number-knower level of both groups, suggesting a comparable level of number knowledge in children with WS and their control group, *t*(18) = 1.5, *ns*, Cohen’s *d* = .69.

### Language screening

#### Comparison WS-DS

Participants with WS yielded significantly higher average raw scores (mean score = 27.7) compared to participants with DS (mean score = 18), even when differences in mental age were controlled, *F*(1, 22) = 4.0, *p* = .03 (one-tailed), *η*_*p*_^2^ = .15. In line with previous findings, our results confirm advanced performance of participants with WS as opposed to participants with DS in the language screening test.

#### Comparison DS-CG_DS_

The language screening revealed significant differences between DS and TD participants, with significantly higher performance of TD children (mean score = 23.3) compared to participants with DS (mean score = 18), *t*(14) = 2.2, *p* = .04, Cohen’s *d* = 0.7. That means that individuals with DS displayed poorer language abilities as would be expected on the basis of their nonverbal mental age.

#### Comparison WS-CG_WS_

No statistically significant differences were obtained with respect to the language screening task for individuals with WS (mean score = 27.7) and TD participants (mean score = 26.9), *t*(9) = 0.4, *ns*, Cohen’s *d* = 0.2).

### Quantifier comprehension

#### Comparison WS-DS

**Fig 1:**
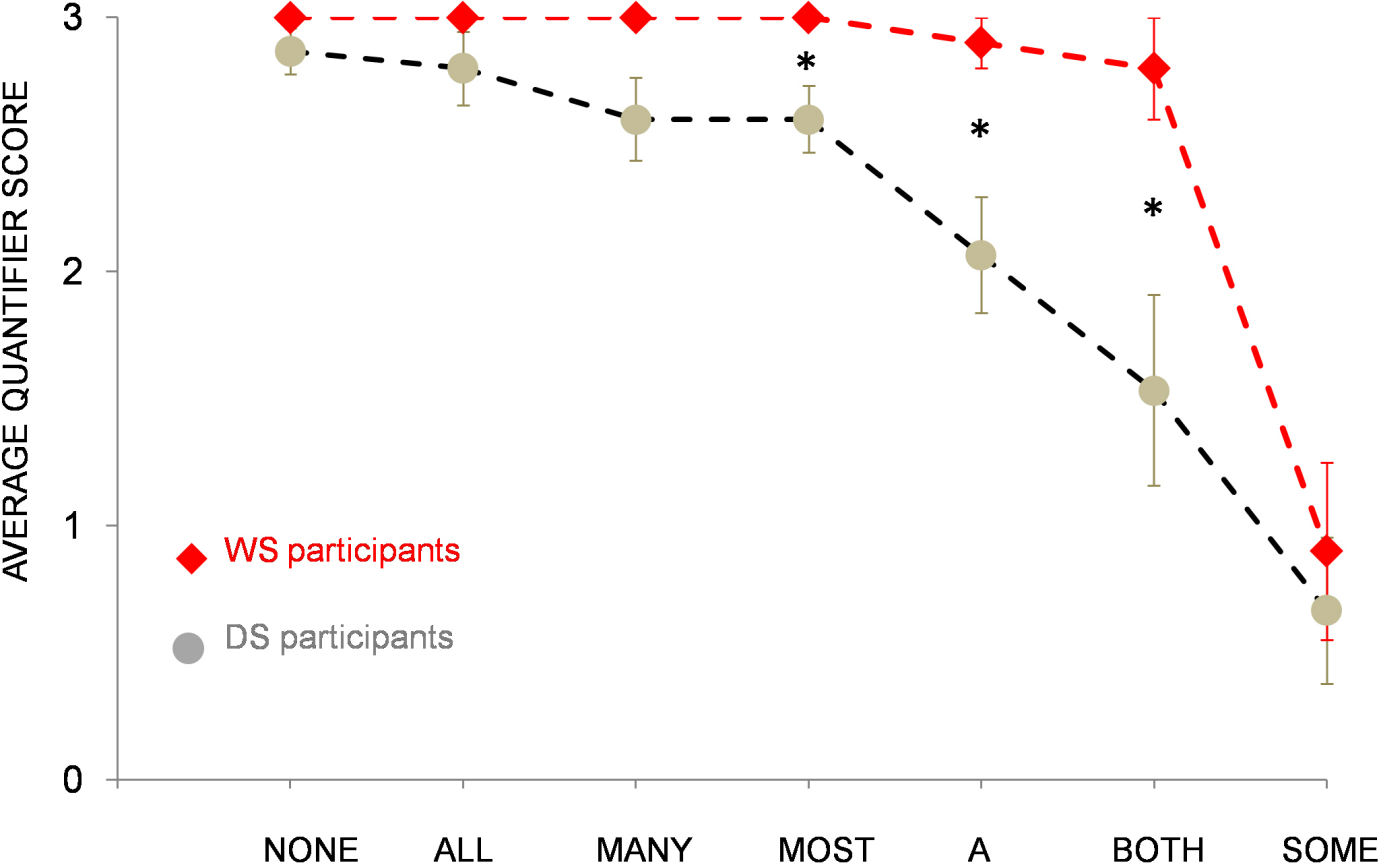
Comparison WS-DS. Average scores of participants with WS and DS for individual quantifiers. Error bars represent standard errors of the mean.

Overall, participants with WS displayed significantly higher average quantifier scores (mean score = 2.7) compared to DS participants (mean score = 2.2), even when mental age was controlled, *F*(1, 22) = 5.35, *p* = .03, *η*_*p*_^2^ = .20. When focusing on individual quantifiers, results revealed numerically higher average quantifier scores for children with WS compared to children with DS for all quantifiers (Fig 1). Differences between individual quantifiers were significant for the quantifiers *most* (*p* = .02), *a* (*p* = .01), and *both* (*p* = .02). Despite quantitative differences, results indicate a similar pattern of quantifier knowledge. That is, both participants with WS and DS show good comprehension of the quantifiers *none*, *all*, *many*, and *most*. Both groups also appear to have specific difficulties in comprehending the quantifier *some*.

#### Comparison DS-CG_DS_

**Fig 2:**
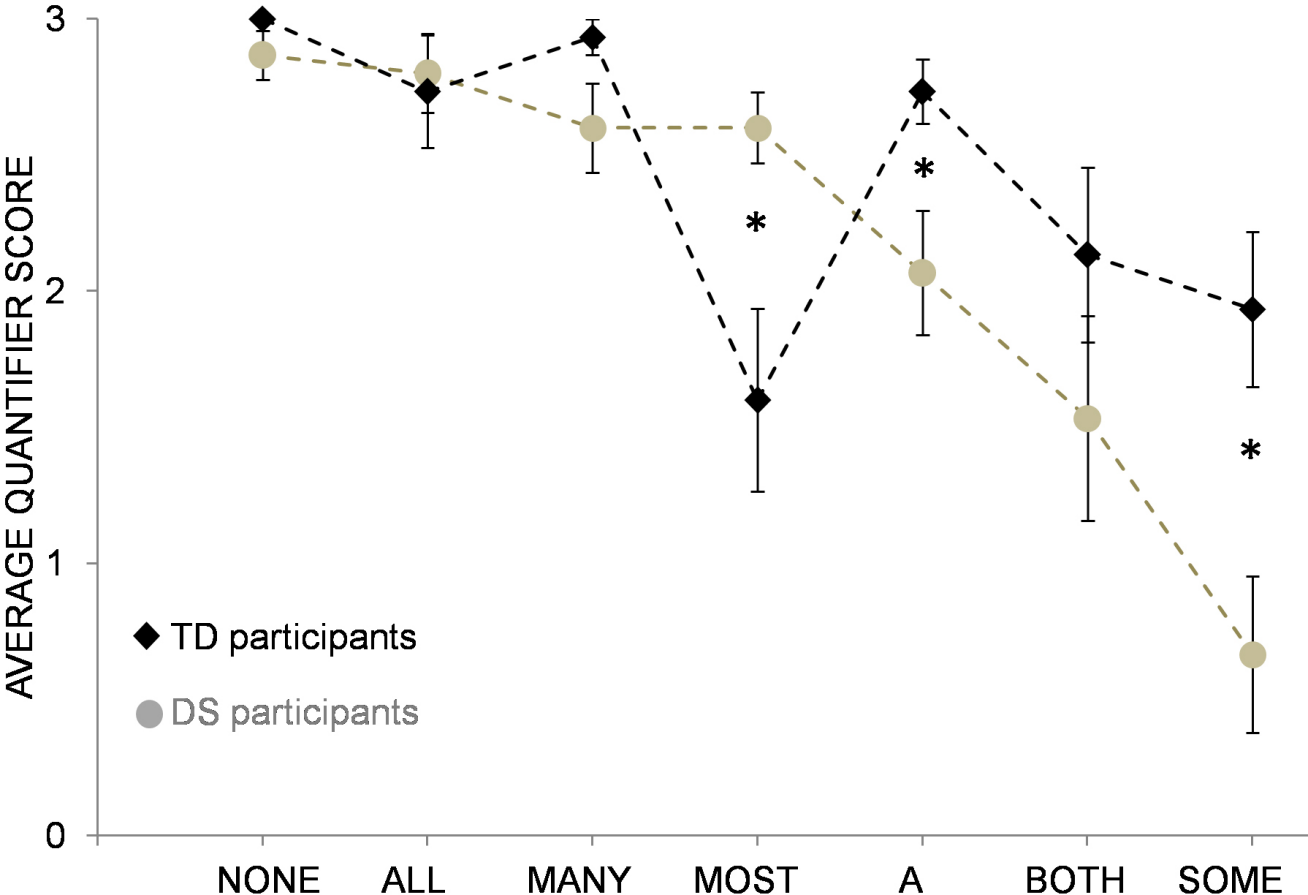
Comparison DS-TD. Average scores of participants with DS and TD children for individual quantifiers. Error bars represent standard errors of the mean.

Overall, quantifier scores differed significantly between children with DS and TD children; *t*(28) = 2.1, *p* < .05, Cohen’s *d* = 0.8, with higher overall performance of TD children (mean score = 2.4) compared to children with DS (mean score = 2.2).When focusing on individual quantifiers, TD children showed better comprehension of the quantifiers *a* (*t*(21) = 2.6, *p* = .02, Cohen’s *d* = 1.1) and *some* (*t*(28) = 3.1, *p* = .01, Cohen’s *d* = 1.2, see Fig 2). Participants with DS, on the other hand, displayed significantly better comprehension of the quantifier *most*, *t*(19) = 2.8, *p* = .01, Cohen’s *d* = 1.3. In general, the pattern of quantifier knowledge seems to differ between individuals with DS and TD children (as can be seen from the differently shaped curves of quantifier performance in Fig 2).

#### Comparison WS-CG_WS_

**Fig 3:**
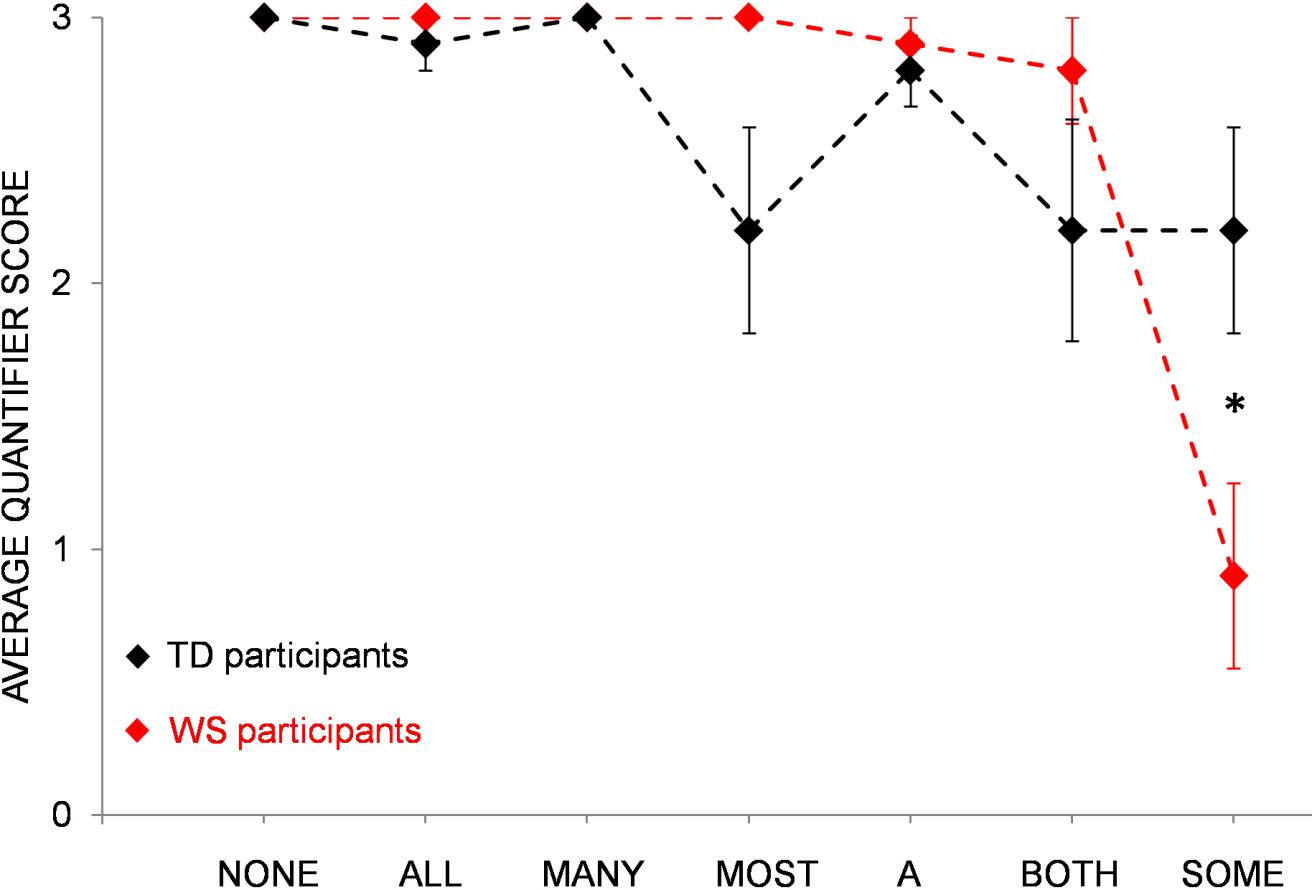
Comparison WS-TD. Average scores of participants with WS and TD children for individual quantifiers. Error bars represent standard errors of the mean.

Quantifier scores did not differ between children with WS (mean score = 2.7) and TD children (mean score = 2.6), *t*(18) = .72, *ns*, Cohen’s *d* = .34, suggesting comparable levels of quantifier comprehension overall. However, when analyzing individual quantifiers separately, children with WS showed significantly worse comprehension of the quantifier *some* compared to TD controls; *t*(18) = 2.5,*p* = .02, Cohen’s *d* = 1.2. There was also a trend for a better comprehension of the quantifier *most* in participants with WS, *t*(18) = 2.1,*p* = .05, Cohen’s *d* = 0.9 (see Fig 3). Quantifier performance of individuals with WS thus seems to deviate somewhat from typically developing controls when individual quantifiers are taken into account.

### Links between language abilities, number knowledge and quantifier comprehension

In order to examine linguistic skills and number knowledge as potential predictors for quantifier knowledge, a linear regression analysis was performed. The average quantifier score for all tested quantifiers was included as the dependent variable. All of the participating groups (i.e. individuals with WS, DS and the respective control groups) were included in the analysis. To take into account differences in cognitive abilities, mental age was also included as a predictor. Results revealed that language abilities had a significant influence on quantifier score (standardized *β* = .63, *p* = .001), whereas number skills (standardized *β* = .02, *ns*) and mental age (standardized *β* = -.10, *ns*) did not. These results confirm that quantifier skills seem to be linked to language rather than to numerical abilities.

Finally, we examined whether linguistic abilities mediated DS and WS children’s quantifier skills. While the type of syndrome (i.e. DS vs. WS) had a significant effect on quantifier performance (*β* = .50, *p* = .002), this effect disappeared when language abilities were added to the model (*β* = .27, *ns*). A Sobel test further revealed a significant reduction of the effect of syndrome type on quantifier skills after including language abilities, z = 1.90, *p* <. 05, suggesting that linguistic skills indeed mediate quantifier knowledge of children with DS and WS.

## Summary

Overall, our results demonstrate that children with WS outperform children with DS regarding quantifier comprehension, even when their mental age is controlled and their number skills are comparable. Since children with WS are also better in a language screening test (in line with previous findings demonstrating advanced language skills in individuals with WS), these results suggest that superior quantifier comprehension is linked to language rather than to numerical abilities. Comparisons with a typically developing control group further corroborate this finding: While participants with WS obtain linguistic and quantifier comprehension scores that are comparable to those of typically developing children of the same mental age, participants with DS display both poorer language skills and quantifier knowledge. The impact of language abilities on quantifier knowledge was also confirmed by a linear regression analysis: Only language but not number skills significantly predicted performance on the quantifier task, suggesting that language abilities play an important role in quantifier comprehension.

## General discussion

To examine whether quantifier knowledge is linked to language or numerical abilities, we investigated two groups of participants that differ in their language skills while we find their numerical abilities to be comparable. In the present study, number skills were assessed by a Give-*n* task. Although it has to be acknowledged that performance on this task was rather high overall, no differences were detected across the different groups of participants, suggesting comparable levels of number skills in children and adolescents with WS and DS as well as their control groups. This finding is in line with previous studies showing that number skills in individuals with DS and WS are rather poor compared to their chronological age and only at the level of their much younger mental age-matched controls [20,21,31]. It should be noted, however, that a direct comparison of number skills in individuals with DS and WS is currently missing (for one exception see [32]). Unlike in our study, Paterson and colleagues found number skills of WS participants to be weaker than those of DS participants [32]. However, Paterson and colleagues did not include cardinal number knowledge in their testing battery, which could be one reason for the observed difference. More crucially, if Paterson and colleagues’ findings are correct, this would even strengthen our results regarding the role of language abilities in quantifier knowledge. That is, in case quantifier comprehension was based on number skills instead of language abilities, then DS participants with superior number skills should also display better quantifier comprehension compared to participants with WS. However, our results reveal the exact opposite pattern: Participants with WS obtained higher levels of quantifier knowledge than individuals with DS. Thus, even if our participants with DS were to have better number skills compared to individuals with WS, these skills are unlikely to determine quantifier comprehension.

Unlike comparable levels of cardinal number knowledge, participants with WS and DS differed significantly with respect to their language abilities: Children with WS outperformed children with DS in a standardized language screening, confirming superior performance of individuals with WS compared to DS participants in the verbal domain [18,23]. Moreover, whereas WS participants’ linguistic skills were on par with their mental age-matched controls, participants with DS performed worse than mental age-matched controls in the language task. These findings are in line with previous results demonstrating impaired language production and comprehension skills in children and adolescents with DS [23,33,34].

For the Give-quantifier task, our results further revealed that individuals with WS outperformed individuals with DS, suggesting that linguistic skills rather than numerical knowledge contribute to quantifier comprehension. In line with this explanation, DS participants’ poorer performance on the Give-quantifier task cannot be explained by a general deficit in task comprehension. Children with DS were able to understand the task as can be seen from their high performance level for some of the quantifiers (e.g. most of the children were able to correctly give eight fruit tokens as a response to the quantifier *all*). Despite their overall comprehension of the task, individuals with DS performed worse than those with WS, stressing the role of linguistic skills in quantifier comprehension. Additionally, our results revealed that WS participants whose language abilities were equivalent to a TD control group also displayed a comparable level of quantifier knowledge. Individuals with DS, on the other hand, performed worse than TD children on both the language screening and the Give-quantifier task. While impairments of language skills in individuals with DS have been reported previously [23,33], here we provide first evidence of concomitant deficits in quantifier comprehension. DS participants’ lower verbal skills thus seem to go hand in hand with poorer quantifier knowledge.

The impact of linguistic abilities was also confirmed by a linear regression analysis in which language abilities but not number knowledge significantly predicted quantifier comprehension. Although these results suggest that linguistic rather than numerical skills are important, they do not imply that numerical skills are entirely irrelevant for quantifier comprehension. As previous work demonstrates, there seem to be close links between number knowledge and quantifier skills in different age-groups and populations [12–14]. Yet, when directly compared, language abilities appear to be more critical than number skills. These findings raise the question which specific linguistic (sub-)skills may contribute to quantifier knowledge. Various candidates have been proposed in this context, including syntactic, semantic, and pragmatic abilities [35,36]. While the language screening administered in our study mainly served to verify divergent linguistic profiles of individuals with WS and DS, future studies have to determine the exact contributions of different linguistic skills to quantifier knowledge in more detail.

### Individual quantifiers

In addition to average performance on the Give-quantifier task, we also analyzed children’s comprehension of individual quantifiers. Although participants with WS outperformed participants with DS with respect to their overall level of quantifier knowledge, the pattern of individual quantifier comprehension appears to be comparable between the two groups. That is, both groups displayed similar strengths and weaknesses regarding quantifier knowledge (e.g. good comprehension of the quantifier *all*, but poor comprehension of the quantifier *some*), resulting in a similarly shaped curve of individual quantifier performance, see Fig 1). When compared to each other, both control groups of typically developing children also showed similar levels of quantifier comprehension, with high levels of performance for exact quantifiers like *all*, *none*, or *a*, and somewhat weaker performance for vague quantifiers like *some* or *most*. This pattern of acquisition matches previous findings demonstrating that ‘totality’ quantifiers like *all* or *none* are acquired earlier than ‘partiality’ quantifiers like *some* [37]. The same is true for TD children’s difficulties in comprehending the quantifier *most*. As has been shown previously, full comprehension of the expression *most* proves to be difficult and even 7-year-old children may not be entirely competent with this quantifier [38]. Thus, while TD children’s quantifier knowledge is in accordance with other findings, quantifier comprehension of individuals with WS and DS appears to be somewhat different. In particular, individuals with WS and DS were significantly worse in comprehending the quantifier *some* when compared to controls. It is possible that the observed discrepancy is due to differences in quantifier type. That is, while quantifiers like *none* or *both* require an exact interpretation (i.e., they denote 0 or 2, respectively), quantifiers like *many* or *some* can map to a range of quantities. Moreover, ‘vague’ quantifiers like *some* can be interpreted in a semantic/logical way or in a pragmatic manner [38]. Although it is logically acceptable to say that there are *some* of the oranges in the bowl in case that all of the oranges are in there, it violates pragmatic principles (e.g. the maxim of informativeness) [1]. Thus, if a person knows that all of the oranges are in the bowl, only the pragmatically correct description *all* would be sufficiently informative. Conversely, the pragmatically correct use of the quantifier *some* entails an upper bounded interpretation, implying that *not all* of the oranges are in the box. While German-speaking adults adhered to an upper bounded interpretation of the quantifier *some* (i.e. they gave a range of 2- to 6 of 8 items but never more [14]), children with WS and DS did not. That is, they frequently gave 8 (= all) items as a response to the quantifier *some*, even significantly more often than TD children matched on mental age. This finding indicates that participants with WS and DS may have specific problems in understanding pragmatic restrictions of quantifiers. However, if pragmatic factors are indeed problematic for individuals with WS and DS, why do they show advanced performance in comprehending the quantifier *most* compared to typically developing controls? On one possibility, participants with WS and DS may have benefitted from longer experience with this quantifier due to their higher chronological age. Alternatively, their advanced performance can be explained by the lack of upper boundedness as an evaluation criterion for the quantifier *most* (i.e. a range of 5- to 8 items was considered a correct response, see Table 2). In line with this assumption, DS and WS participants’ superior performance for vague quantifiers like *most* or *many* seems to disappear when responses are evaluated in an upper bounded way (i.e. by excluding 8 (= all) items as a correct response). For the quantifier *most*, for instance, DS children’s scores decrease from 2.6 to 0.3 (CG_DS_: 0.9), and WS children’s scores decrease from 3.0 to 0.9 (CG_WS_: 1.3). While these findings suggest that both groups of individuals with WS and DS may have problems with pragmatic restrictions of quantifiers, it should be stressed that this pattern was not unprecedented. That is, when tested in the same experiment, around 20 percent of the German adult speakers also gave 8 (i.e. *all*) items as a response for the quantifier *most*, demonstrating that the Give-quantifier task we employed here does not necessarily elicit an upper bounded response [14]. On the basis of these findings, we considered it counterintuitive to judge children’s responses as incorrect when they were in line with German adults’ judgments in the very same task [14]. However, even when vague quantifiers were evaluated in an upper bounded way, participants with WS still outperformed individuals with DS, suggesting that adherence to pragmatic principles is not the main ingredient in the differences observed, *F*(1, 22) = 3.97, *p* = .03 (one-tailed), *η*_*p*_^2^ = .15.

Apart from vague quantifiers, individuals with DS also had problems with exact quantifiers when compared to participants with WS and TD children. That is, children with DS performed significantly worse than children with WS and TD controls in comprehending the quantifiers *a* and *both*. This finding is particularly striking because all of the participants with DS were at least two-knowers and thus able to reliably identify quantities of one and two. However, while they were successful at giving one or two tokens as a response during the Give-*n* task, they often failed when asked to give the same number of items as a response during the Give-quantifier task. On one possibility, the Give-quantifier task may have been more complex than the Give-n task due to the different fruit kinds that the participants had to differentiate. However, since participants with DS had no problems in differentiating the various types of fruit when asked for other quantifiers, this explanation is unlikely to account for the observed difficulties. As for the quantifier *eine* (a), it is possible that DS participants’ behavior was affected by the similar sounding quantifier *einige* (some) which was also assessed during the Give-quantifier task. However, since only participants without any hearing problems were included in the sample, this explanation is rather unlikely. Alternatively, the partitive construction which was exclusively used in the Give-quantifier task but not in the Give-*n* task may have affected DS children’s performance. In particular, the obligatory plural form of the partitive construction (i.e. *a* of the X*s*) may have confused children with DS who then considered one single token an inappropriate response to a plural request. While the exact reasons for DS participants’ response patterns remain open, our results demonstrate that individuals with DS do not only exhibit problems in comprehending vague but also exact quantifiers when compared to TD children as well as to individuals with WS.

## Conclusions

In order to disentangle the specific contributions of linguistic vs. number skills in quantifier comprehension, we tested participants with DS and WS who display comparable numerical skills but who differ in their language abilities. We reasoned that if number skills are essential for comprehending quantifiers, quantifier knowledge should be equivalent in children and adolescents with DS and WS. Conversely, if linguistic skills are more important for quantifier comprehension, we expected participants with WS to outperform participants with DS due to their advanced language skills. Participants with WS indeed demonstrated superior language abilities and also better levels of quantifier comprehension compared to participants with DS. In addition, language abilities but not number skills significantly predicted quantifier knowledge in a linear regression analysis. While the precise contribution of language remains to be determined, our results provide evidence that language abilities rather than numerical skills are essential for quantifier comprehension.

## Supporting information

S1 File Raw data. (CSV)

## References

[1] Grice HP. Logic and conversation. 1975. 1975;41–58.

[2] Cheng D, Zhou A, Yu X, Chen C, Jia J, Zhou X. Quantifier processing can be dissociated from numerical processing: Evidence from semantic dementia patients. Neuropsychologia. 2013;51(11):2172–83. Available from: https://doi.org/10.1016/j.neuropsychologia.2013.07.003 23867350 10.1016/j.neuropsychologia.2013.07.003

[3] Katsos N, Roqueta CA, Estevan RAC, Cummins C. Are children with Specific Language Impairment competent with the pragmatics and logic of quantification? Cognition. 2011;119(1):43–57. Available from: https://doi.org/10.1016/j.cognition.2010.12.004 21237449 10.1016/j.cognition.2010.12.004

[4] Cappelletti M, Butterworth B, Kopelman M. Numeracy skills in patients with degenerative disorders and focal brain lesions: A neuropsychological investigation. Neuropsychology. 2012;26(1):1–19. doi: 10.1037/a0026328 22122516 10.1037/a0026328 PMC3248328

[5] McMillan CT, Clark R, Moore P, Devita C, Grossman M. Neural basis for generalized quantifier comprehension. Neuropsychologia. 2005;43(12):1729–37. doi: 10.1016/j.neuropsychologia.2005.02.012 16154448 10.1016/j.neuropsychologia.2005.02.012

[6] Pietroski P, Lidz J, Lidz J, Hunter T, Hunter T, et al The meaning of “Most”: semantics,numerosity, and psychology. Mind Lang. 2009;24(5):554–85.

[7] Shikhare S, Heim S, Klein E, Huber S, Willmes K. Processing of Numerical and Proportional Quantifiers. Cogn Sci. 2015;39(7):1504–36. doi: 10.1111/cogs.12219 25631283 10.1111/cogs.12219

[8] Troiani V, Peelle JE, Clark R, Grossman M. Is it logical to count on quantifiers? Dissociable neural networks underlying numerical and logical quantifiers. Neuropsychologia. 2009;47(1):104–11. doi: 10.1016/j.neuropsychologia.2008.08.015 18789346 10.1016/j.neuropsychologia.2008.08.015 PMC2637397

[9] Olm CA, McMillan CT, Spotorno N, Clark R, Grossman M. The relative contributions of frontal and parietal cortex for generalized quantifier comprehension. Front Hum Neurosci. 2014;8(August):1–8. Available from: http://journal.frontiersin.org/article/10.3389/fnhum.2014.00610/abstract 25147520 10.3389/fnhum.2014.00610 PMC4124462

[10] Wei W, Chen C, Yang T, Zhang H, Zhou X. Dissociated neural correlates of quantity processing of quantifiers, numbers, and numerosities. Hum Brain Mapp. 2014;35(2):444–54. doi: 10.1002/hbm.22190 23019128 10.1002/hbm.22190 PMC6869002

[11] Hodges JR, Patterson K, Oxbury S, Funnell E. Semantic dementia: Progressive fluent aphasia with temporal lobe atrophy. Brain. 1992;115(6):1783–806. 1486461 10.1093/brain/115.6.1783

[12] Cappelletti M, Butterworth B, Kopelman M. The understanding of quantifiers in semantic dementia: A single-case study. Neurocase. 2006;12(3):136–45. doi: 10.1080/13554790600598782 16801149 10.1080/13554790600598782 PMC2567819

[13] Barner D, Chow K, Yang SJ. Finding one’s meaning: A test of the relation between quantifiers and integers in language development. Cogn Psychol. 2009;58(2):195–219. Available from: https://doi.org/10.1016/j.cogpsych.2008.07.001 18799157 10.1016/j.cogpsych.2008.07.001

[14] Dolscheid S, Winter C, Ostrowski L, Penke M. The many ways quantifiers count: Children’s quantifier comprehension and cardinal number knowledge are not exclusively related. Cogn Dev. 2017;44.

[15] Donlan C, Cowan R, Newton EJ, Lloyd D. The role of language in mathematical development: Evidence from children with specific language impairments. Cognition. 2007;103(1):23–33. doi: 10.1016/j.cognition.2006.02.007 16581052 10.1016/j.cognition.2006.02.007

[16] Durkin K, Pearl PL, Conti-Ramsden G. Severity of specific language impairment predicts delayed development in number skills. Front Psychol. 2013;4(SEP):1–10. 24027548 10.3389/fpsyg.2013.00581 PMC3759789

[17] Francke U. Williams-Beuren syndrome: Genes and mechanisms. Hum Mol Genet. 1999;8(10):1947–54. 10469848 10.1093/hmg/8.10.1947

[18] Bellugi U, Lichtenberger L, Jones W, Lai Z, St. George M. I. The Neurocognitive Profile of Williams Syndrome: A Complex Pattern of Strengths and Weaknesses. J Cogn Neurosci. 2000;12(supplement 1):7–29. Available from: http://www.mitpressjournals.org/doi/10.1162/089892900561959 10953231 10.1162/089892900561959

[19] Meyer-Lindenberg A, Mervis CB, Faith Berman K. Neural mechanisms in Williams syndrome: A unique window to genetic influences on cognition and behaviour. Nat Rev Neurosci. 2006;7(5):380–93. doi: 10.1038/nrn1906 16760918 10.1038/nrn1906

[20] Ansari D, Donlan C, Thomas MSC, Ewing SA, Peen T, Karmiloff-Smith A. What makes counting count? Verbal and visuo-spatial contributions to typical and atypical number development. J Exp Child Psychol. 2003;85(1):50–62. 12742762 10.1016/s0022-0965(03)00026-2

[21] Brigstocke S, Hulme C, Nye J. Number and arithmetic skills in children with Down syndrome. Down Syndr Res Pract. 2008;74–78.

[22] O’Hearn K, Landau B. Mathematical skill in individuals with Williams syndrome: Evidence from a standardized mathematics battery. Brain Cogn. 2007;64(3):238–46. doi: 10.1016/j.bandc.2007.03.005 17482333 10.1016/j.bandc.2007.03.005 PMC2104493

[23] Bellugi U, Bihrle A, Jernigan TL, Trauner D, Doherty S. Neuropsychological, neurological, and neuroanatomical profile of Williams syndrome. Am J Med Genet Suppl. 1990;6:115–25. 2144426 10.1002/ajmg.1320370621

[24] Krause M, Penke M. Inflectional morphology in German Williams syndrome. Brain Cogn. 2002;48(2–3):410–413. Available from: http://europepmc.org/abstract/MED/12030478 12030478

[25] Chapman R, Seung H, Schwartz SE, Bird EK. Predicting Language Production in Children and Adolescents With Down Syndrome: The Role of Comprehension. J Speech, Lang Hear Res. 2000;43:340–50. 10757688 10.1044/jslhr.4302.340

[26] Abbeduto L, Murphy MM, Cawthon SW, Richmond EK, Weissman MD, Karadottir S, et al Receptive Language Skills of Adolescents and Young Adults With Down or Fragile X Syndrome. Am J Ment Retard. 2003;108(3):149 Available from: http://www.aaiddjournals.org/doi/abs/10.1352/0895-8017(2003)108%3C0149:RLSOAA%3E2.0.CO;2 12691594 10.1352/0895-8017(2003)108<0149:RLSOAA>2.0.CO;2

[27] Tellegen PJ, Laros JA, Petermann F. SON-R 2 1/2-7: non-verbaler Intelligenztest; Testmanual mit deutscher Normierung und Validierung. Hogrefe; 2007.

[28] Grimm H, Aktas M. SSV Sprachscreening für das Vorschulalter: Kurzform des SETK 3–5. Hogrefe Verlag für Psychologie; 2003.

[29] Wynn K. Issues Concerning a Nativist Theory of Numerical Knowledge. Mind Lang. 1992;7(4):367–81.

[30] Sarnecka BW, Carey S. How counting represents number: What children must learn and when they learn it. Cognition. 2008;108(3):662–74. doi: 10.1016/j.cognition.2008.05.007 18572155 10.1016/j.cognition.2008.05.007

[31] Sella F, Lanfranchi S, Zorzi M. Enumeration skills in Down syndrome. Res Dev Disabil. 2013;34(11):3798–9806. Available from: https://doi.org/10.1016/j.ridd.2013.07.038 24025435 10.1016/j.ridd.2013.07.038

[32] Paterson SJ, Girelli L, Butterworth B, Karmiloff-Smith A. Are numerical impairments syndrome specific? Evidence from Williams syndrome and Down’s syndrome. J Child Psychol Psychiatry Allied Discip. 2006;47(2):190–204. 10.1111/j.1469-7610.2005.01460.x 16423150

[33] Chapman R, Hesketh L. Language, cognition, and short-term memory in individuals with Down syndrome. Down Syndr Res Pract. 2001;7(1):1–7. Available from: https://www.down-syndrome.org/reviews/108/10.3104/reviews.108 11706807

[34] Witecy B, Penke M. Das Verhältnis von Sprache und Kognition bei deutschsprachigen Kindern und Jugendlichen mit. Sprache, Stimme, Gehör. 2016;93–9.

[35] Bloom P, Wynn K. Linguistic cues in the acquisition of number words. J Child Lang. 1997;24(3):511–33. 9519584 10.1017/s0305000997003188

[36] Carey S. Bootstrapping & the origin of concepts. Daedalus. 2004;133(1):59–68.

[37] Katsos N, Cummins C, Ezeizabarrena M-J, Gavarró A, Kraljević JK, Hrzica G, et al Cross-linguistic patterns in the acquisition of quantifiers. Proc Natl Acad Sci. 2016;113(33):9244–9. doi: 10.1073/pnas.1601341113 27482119 10.1073/pnas.1601341113 PMC4995931

[38] Katsos N, Bishop DVM. Pragmatic tolerance: Implications for the acquisition of informativeness and implicature. Cognition. 2011;120(1):67–81. doi: 10.1016/j.cognition.2011.02.015 21429481 10.1016/j.cognition.2011.02.015 PMC3104140

